# Xenon Exerts Neuroprotective Effects on Kainic Acid-Induced Acute Generalized Seizures in Rats *via* Increased Autophagy

**DOI:** 10.3389/fncel.2020.582872

**Published:** 2020-10-06

**Authors:** Wei Zhu, Jianguo Zhu, Shengfa Zhao, Jieqing Li, Dianjun Hou, Yurong Zhang, Hongliu Sun

**Affiliations:** ^1^Institute of Radiation Medicine, Shandong Academy of Medical Sciences, Shandong First Medical University, Jinan, China; ^2^Juxian People’s Hospital, Linyi, China; ^3^School of Pharmaceutical Sciences, Binzhou Medical University, Yantai, China

**Keywords:** seizure, autophagy, neurodegeneration, apoptosis, xenon inhalation

## Abstract

Xenon has been shown to have neuroprotective effects and is clinically used as a favorable safe inhalation anesthetic. We previously confirmed the neuroprotective effects of xenon treatment in epileptic animals. However, the mechanism underlying these protective effects remains unclear. We aimed to assess the effects of xenon inhalation on autophagy in neuronal injury induced by acute generalized seizures. Kainic acid (KA) was injected into the lateral ventricle of male Sprague–Dawley rats to induce acute generalized seizures. Next, the rats were treated *via* inhalation of a 70% xenon/21% oxygen/9% nitrogen mixture for 60 min immediately after KA administration. The control group was treated *via* inhalation of a 79% nitrogen/21% oxygen mixture. Subsequently, two inhibitors (3-methyladenine or bafilomycin A_1_) or an autophagy inducer (rapamycin) were administered, respectively, before KA and xenon administration to determine the role of autophagy in the protective effects of xenon. The levels of apoptosis, neuronal injury, and autophagy were determined in all the rats. Xenon inhalation significantly attenuated the severity of the seizure-induced neuronal injury. Increased autophagy accompanied this inhibitive effect. Autophagy inhibition eliminated these xenon neuroprotective effects. A simulation of autophagy using rapamycin recapitulated xenon’s protective effects on KA-induced acute generalized seizures in the rats. These findings confirmed that xenon exerts strong neuroprotective effects in KA-induced acute generalized seizures. Further, they indicate that increased autophagy may underlie the protective effects of xenon. Therefore, xenon and autophagy inducers may be useful clinical options for their neuroprotective effects in epileptic seizures.

## Introduction

Epilepsy is a common neurological disease that affects 0.5–1% of the worldwide population with potentially serious consequences, including neuronal injury and cognitive defects. There are currently available drug and surgical treatment options for epilepsy and associated cognitive defects. However, they have several limitations, including drug resistance, serious side effects, and recrudescence (Schmidt and Löscher, 2005; Chen et al., 2018). Therefore, there is a need for more safe and effective therapeutic strategies for treating epileptic seizures, as well as seizure-induced neuronal injury and cognitive defects.

Xenon is clinically used as a safe anesthetic agent and is popular due to having almost no side effects. It has been recently receiving increased attention due to its superior neuroprotective effects, which have been confirmed in Alzheimer’s disease (Lavaur et al., 2016a,b) and in ischemia/reperfusion injury (Cattano et al., 2011; Yang T. et al., 2012; Yang et al., 2014; Metaxa et al., 2014). Moreover, xenon treatment has been shown to exert similar neuroprotective effects and ameliorate cognitive impairment in intrauterine (Yang Y. W. et al., 2012) and neonatal asphyxia (Luo et al., 2008). Further, we previously demonstrated it had anti-epileptic and neuroprotective effects in seizure-induced neuronal injury (Zhang et al., 2019a,b). Further studies are required to explore the underlying mechanisms of xenon treatment effects and reveal potential targets for neuronal protection in epilepsy and seizure.

There is a correlation of epilepsy- and seizure-induced neuronal injury with over-excitation induced by increased glutamate levels (Malinska et al., 2010; Kovac et al., 2016; Liang et al., 2019). Glutamate metabolism deficits may cause reactive oxygen species (ROS) production. Furthermore, oxidative stress induced by ROS accumulation may lead to neuronal injury (Shekh-Ahmad et al., 2019; Zhang et al., 2020b) and induce autophagy (Signorelli et al., 2019). Therefore, there is a close correlation of autophagy with an excitotoxicity-induced neuronal injury during epileptic seizures. However, autophagy has been shown to promote both cell survival and death, which depends on the different psychological or pathological environments (Zhang et al., 2013, 2014; Feng et al., 2020). Although there have been inconsistent findings regarding the role of autophagy in cell injury, autophagy has been suggested to have protective effects in epileptic animals (Jain et al., 2016; Ni et al., 2016).

Xenon can attenuate over-excitation by the regulation of glutamate metabolism, through inhibiting glutamate uptake and efflux (Lavaur et al., 2016a,b). Given the initiating role of over-excitation in neuronal injury and autophagy, as well as the effect of autophagy on the seizure-induced neuronal injury, we hypothesized that autophagy is closely related to the neuroprotective effects of xenon.

We aimed to evaluate the effect of xenon inhalation on seizure-induced neuronal injury after KA administration. Furthermore, we aimed to assess the role of autophagy in the protective effect of xenon, as well as to explore the role of autophagy regulation in KA-induced acute generalized seizures.

## Materials and Methods

### Animals and Surgery

We conducted the experiments using male Sprague–Dawley rats (240–260 g, Certificate No. SCXK2014-0006; Jinan Jinfeng Experimental Animal Company Limited, China). All experiment protocols were approved by the Binzhou Medical University Animal Experimentation Committee (Approval No. 2018002) and comply with the National Institutes of Health Guide for the Care and Use of Laboratory Animal (NIH Publications No. 80-23, revised 1996). We made maximum effort to minimize the number of rats used and to attenuate suffering. The rats were raised in individual cages where water and food were provided *ad libitum*. All animal experiments, including surgery, drug treatment, and xenon inhalation, were performed between 09:00 h and 17:00 h.

The rats were anesthetized using sodium pentobarbital (50 mg/kg, intraperitoneal injection, CAS, 57-33-0, Xiya Reagent, China) and fixed on a stereotactic apparatus (Anhui Zheng Hua Biological Instrument Equipment Company Limited, China). As previously described (Zhang et al., 2020a), stainless steel cannulas (Reward, China) were implanted into the right lateral cerebral ventricle (AP: −1.8 mm, ML: −0.96 mm, and DV: −3.8 mm). The rats were allowed 7 days for recovery.

### KA-Induced Acute Generalized Seizure

Acute generalized seizures were induced through KA treatment (3.25 × 10^–3^ mg/kg, 1.25 mg/ml, CAS, 58002-62-3, Sigma, USA), which was injected into the lateral ventricle *via* the implanted cannulas. The seizure severity was assessed Racine’s criteria (Racine, 1972). Immediately after KA treatment, almost all the rats presented with signs of continuous acute generalized seizures (mainly stage 4 or 5). Sixty minutes after KA treatment, diazepam (2 mg/kg, CAS, 439-14-5, Sigma, USA) was intraperitoneally injected to stop the seizures. Finally, the cannula location was histologically verified. We excluded data from rats with inaccurate cannula locations from the analysis.

### Xenon Treatment

KA-treated rats were randomly placed into two transparent resin boxes. Rats in the xenon group were treated with 70% xenon/21% oxygen/9% nitrogen (DaTe Special Gas Limited, China) for 1 h immediately after KA injection (De Deken et al., 2018), while those in the control group were treated with a 21% oxygen/79% nitrogen mixture (Rulin gas Limited, China).

The gas delivery speed (200 ml/min) was controlled by flow regulator valves (DaTe Special Gas Limited, China), which were installed in the gas bottles. The rats remained sober with stable temperatures during the entire xenon treatment. Sixty minutes after KA administration, diazepam was intraperitoneally injected to stop KA-induced seizures. Supplementary Figure 1 presents details of the experimental procedure.

### Drug Administration

The rats were randomly divided into the drug administration groups and the control group. Six hours before xenon treatment, autophagy inhibitors [3-methyladenine (3-MA, 75 μg, CAS, 5142-23-1, Sigma, USA—dissolved in 5 μl saline) or bafilomycin A_1_ (BafA_1_, 200 ng, CAS, 88899-55-2, Sigma, USA) dissolved in dimethyl sulfoxide and 5 μl saline] were injected into the right lateral cerebral ventricle. Rats in the control group were instead treated with 5 μl saline. Subsequently, all the rats were treated with xenon (70% xenon/21% oxygen/9% nitrogen) immediately after KA injection.

One hour before KA treatment, an autophagy inducer (rapamycin, 200 ng, CAS, 53123-88-9, Sigma, USA; dissolved in 5 μl saline) was injected into the right cerebral ventricle while rats in the control group were instead treated with 5 μl saline.

### Western Blot Analysis

As previously described (Zhang et al., 2019c), five rats from each group were randomly anesthetized using sodium pentobarbital (50 mg/kg, intraperitoneal injection, Xiya Reagent, China) at the different time points after KA administration (24 h, 3 days, or 7 days) and culled. Their brains were immediately retrieved and different subregions, including the hippocampus, pyriform cortex (PC), and remaining cortex, were dissected on ice. Protein levels in each sample were measured (P0012, Beyotime Institute of Biotechnology, China) after sonication. Next, proteins with similar concentrations were loaded and separated using 12% sodium dodecyl sulfate-polyacrylamide gels and transferred to polyvinylidene difluoride membranes. After blocking for 1 h using 5% skim milk, the membranes were incubated overnight at 4°C with the following primary antibodies: rabbit polyclonal antibody against caspase-3 (9662, 1:1,000, Cell Signaling Technology, Danvers, MA, USA), rabbit monoclonal antibody against activated caspase-3 (ab2302, 1:1,000, Abcam, USA), mouse monoclonal antibody against B cell lymphoma-2 (Bcl-2, ab32124, 1:1,000, Abcam, USA), Bcl-2-associated X protein (Bax, ab77566, 1:1000, Abcam, USA), sequestosome 1 (SQSTM 1, ab56416, 1:1,000, Abcam, USA), microtubule-associated protein 1 light chain 3 (LC 3, D3U4C, 1:1,000, Cell Signaling Technology, Danvers, MA, USA), and glyceraldehyde-3-phosphate dehydrogenase (GAPDH, AB-P-R 001, 1:2,000, Kangcheng, China). After treatment with horseradish peroxidase-conjugated IgG secondary antibodies, visualized bands were obtained for analysis (Odyssey, LI-COA Biosciences, USA). Protein level differences were presented as the normalized intensity relative to GAPDH.

### Fluoro-Jade B (FJB) Staining

Fluoro-Jade B (FJB) specifically binds to degenerating neurons, and FJB staining is used to evaluate neurodegeneration (Schmued and Hopkins, 2000). From each group, five rats were randomly anesthetized using sodium pentobarbital (50 mg/kg, intraperitoneal injection, Xiya Reagent, China) and perfused. Coronal slices (10 μm) were obtained using a cryostat microtome (CM3050s, Leica, Germany). As previously described (Zhang et al., 2019a,b), we performed the staining procedure (three slices/rat) as per the kit manufacturer’s instructions (AG310, Millipore, USA). Finally, the stained slices were examined under a fluorescent microscope with a 450 nm excitation light (Carl Zeiss AG, Germany), and the number of positive FJB signals (300 × 300 μm vision) in changed subregions was determined and analyzed.

### Immunohistochemistry

At designated time points (24 h, 3 days, and 7 days) after KA treatment, five mice in every group were deeply anesthetized and coronal slices were obtained, as performed in FJB staining. LC3B/DAPI fluorescence staining was performed as per the following protocol: After being treated with rabbit anti-LC3B (1:100; ab48394, Abcam, UK) and washing three times, the sections were incubated in the secondary antibody (FITC, 1:200, EMD Millipore, USA). After three washes, the sections were incubated in DAPI (C1005, Beyotime Institute of Biotechnology, China) for 15 min at room temperature and were finally rinsed three times with 0.01 M PBS for 5 min each.

All fluorescence images were acquired with a laser confocal microscope (Zeiss, Germany) under the same capture conditions and were analyzed using ImageJ V.1.37 software (National Institutes of Health, Bethesda, MD, USA).

### Statistical Analysis

Investigators who obtained all of the data were blinded and the data are presented as the mean ± standard error of the mean (SEM). All statistical analyses were performed using SPSS version 13.0 software (SPSS Inc., Chicago, USA). The nonparametric Mann–Whitney *U* test was used to compare protein levels and the number of positive FJB signals. Statistical significance was set at a *p*-value of < 0.05.

## Results

### Xenon Treatment Reduced Apoptosis and Neuronal Injury With an Accompanying Increase in the Autophagy Level

**Figure 1:**
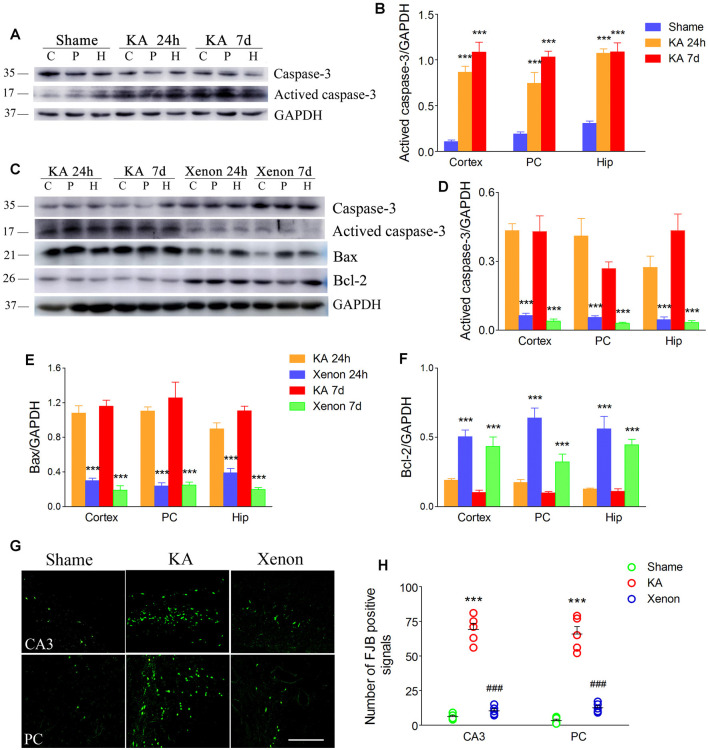
Xenon treatment attenuated apoptosis and neurodegeneration during kainic acid (KA)-induced acute generalized seizures. **(A)** Changes of caspase-3 and activated caspase-3 levels after KA treatment; **(B)** normalized intensity of activated caspase-3 relative to GAPDH; **(C)** changes of caspase-3, activated caspase-3, Bax, and Bcl-2 after xenon inhalation; **(D–F)** normalized intensity of activated caspase-3, Bax and Bcl-2 relative to GAPDH; **(G)** changed positive Fluoro-Jade B (FJB) signals (bar = 100 μm); **(H)** analysis of positive FJB signals. Data are presented as mean ± SEM. Error bars indicate SEM (*n* = 5/group; ***P < 0.001, compared with controls; ^###^*P* < 0.001 compared with KA group; one-way ANOVA). C, cortex except pyriform cortex; P/PC, pyriform cortex; H, hippocampus.

We analyzed apoptosis-related markers after KA administration (*n* = 5 per group). Western blot analysis revealed increased activated caspase-3 levels in the hippocampus and cortex at 24 h and 7 days after KA administration (Figures 1A,B). Further, we compared the apoptosis level between rats treated with and without xenon after KA administration (*n* = 5 per group). We found that xenon treatment significantly attenuated KA-induced changes in apoptosis-related proteins. There was a significant decrease in the immunoreactivity of activated caspase-3 (Figures 1C,D) and Bax (Figures 1C,E) and increases in that of caspase-3 (Figure 1C) and Bcl-2 (Figures 1C,F) in the xenon-treated rats compared with the control rats. Consistent with this, xenon treatment significantly attenuated the increased number of positive FJB signals in the CA3 and PC after KA administration (*n* = 5 per group, Figures 1G,H). These findings indicate that xenon inhalation could prevent apoptosis and neuronal injury associated with KA-induced acute generalized seizures.

**Figure 2:**
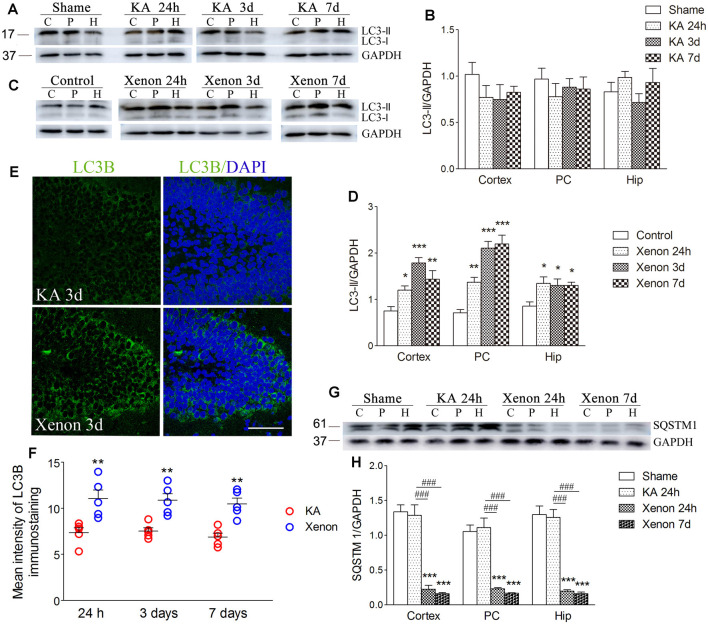
Xenon treatment increased the level of autophagy during KA-induced acute generalized seizures. **(A)** change of LC3 expression after KA administration; **(C)** changes of LC3 expression after xenon treatment; **(B,D)** normalized intensity of LC3-II relative to GAPDH; **(E,F)** immunoreactivity of LC3B; **(G)** the expression of SQSTM1; and **(H)** normalized intensity of SQSTM1 relative to GAPDH. Data are presented as mean ± SEM. Error bars indicate SEM (*n* = 5/group; *P < 0.05, **P < 0.01, and ***P < 0.001, compared with controls; ^###^*P* < 0.001 compared with KA group; one-way ANOVA). C, cortex except pyriform cortex; P/PC, pyriform cortex; H, hippocampus.

Moreover, we investigated changes in the autophagy levels in the different groups (*n* = 5 per group). SQSTM1, LC3-II, and LC3B are considered autophagy markers. After KA injection, there was no significant change in LC3-II/GAPDH levels in the KA group compared with the control group (Figures 2A,B). Notably, xenon treatment significantly elevated the LC3-II/GAPDH ratio in the cortex with the PC removed (*P* = 0.014), PC (*P* = 0.002), and hippocampus (*P* = 0.024, Figures 2C,D) from 24 h after administration. The immunoreactivity of LC3B increased after xenon treatment (Figures 2E,F). There is a negative relationship between SQSTM1 expression and autophagy activity given its usual degradation during autophagy. We observed significantly reduced SQSTM1/GAPDH ratios in the cortex, PC, and hippocampus in the xenon group compared with the KA group, though no significant difference was found between the sham and KA groups (Figures 2G,H). These findings suggest that xenon treatment significantly attenuates neuronal injury with an accompanying enhancement in the autophagy level. Subsequently, to assess whether autophagy promotes or inhibits the neuroprotective effects of xenon, autophagy inhibitors were administrated to xenon-treated rats, while autophagy promoters were administrated to KA-treated rats.

### 3-MA Reversed the Increased Autophagy Level and Xenon Neuroprotective Effect

**Figure 3:**
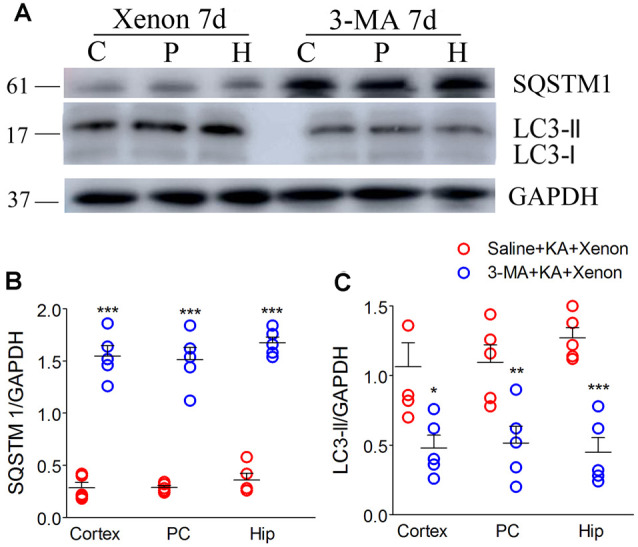
Treatment with 3-methyladenine (3-MA) reversed the increase in autophagic level after xenon inhalation. **(A)** Increased level of SQSTM1 and decreased level of LC3-II; **(B)** normalized intensity of SQSTM1 relative to GAPDH; **(C)** normalized intensity of LC3-II relative to GAPDH. Data are presented as mean ± SEM. Error bars indicate SEM (*n* = 5/group; *P < 0.05, **P < 0.01, and ***P < 0.001, compared with controls, one-way ANOVA). C, cortex except pyriform cortex; P/PC, pyriform cortex; H, hippocampus.

**Figure 4:**
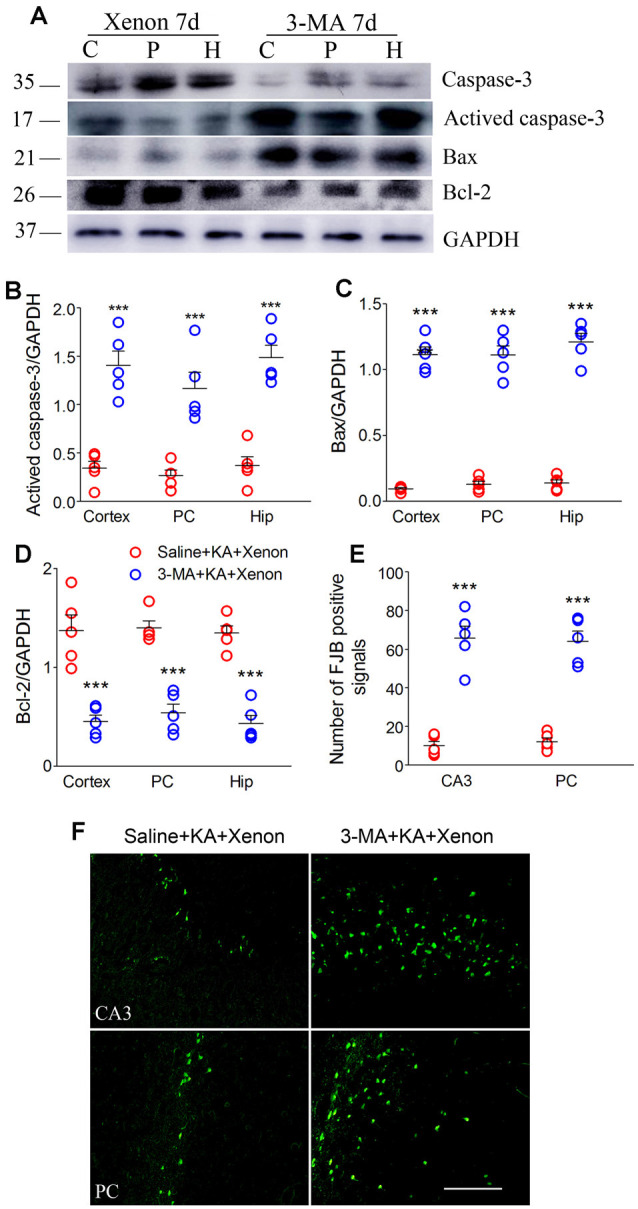
Treatment with 3-MA reversed the attenuated neuronal injury after xenon inhalation. **(A)** Increased level of activated caspase-3 and decreased level of caspase-3; **(B–D)** normalized intensity of activated caspase-3, Bax, and Bcl-2 relative to GAPDH; and **(E,F)** analysis of positive FJB signals (bar = 100 μm). Data are presented as mean ± SEM. Error bars indicate SEM (*n* = 5/group; ***P < 0.001, compared with controls, one-way ANOVA). C, cortex except pyriform cortex; P/PC, pyriform cortex; H, hippocampus.

For autophagy inhibition (Yu et al., 2018), 75 μg 3-MA was injected into the lateral cerebral ventricle 6 h before xenon treatment (3-MA + KA + Xenon group, *n* = 5) while the control rats were injected with saline (Saline + KA + Xenon group, *n* = 5). Compared with the Saline + KA + Xenon group, a significant decrease in the LC3-II/DAPDH ratio (the cortex with the PC removed, *P* = 0.017; the PC, *P* = 0.010; and the hippocampus, *P* < 0.001; Figures 3A,C) and an increase in the SQSTM1/GAPDH ratio (the cortex with the PC removed, *P* < 0.001; the PC, *P* < 0.001; and the hippocampus, *P* < 0.001; Figures 3A,B) in the 3-MA+KA+Xenon group were noted. This indicates that xenon-induced enhanced autophagy was inhibited by 3-MA administration. Moreover, we assessed apoptosis-related proteins. Compared with the controls, the 3-MA-treated rats had a significantly higher activated caspase-3/GAPDH (Figures 4A,B) and Bax/GAPDH ratios (Figures 4A,C), and a significantly lower Bcl-2/GAPDH ratio (Figures 4A,D). Also, compared with the control rats, 3-MA-treated rats had significantly more FJB-positive signals in the CA3 and PC (Figures 4E,F). Taken together, 3-MA reversed the increases in autophagy and prevented the xenon-induced attenuation of apoptosis and neuronal injury.

### BafA1 Similarly Reversed the Enhanced Autophagy and Xenon Neuroprotective Effect

**Figure 5:**
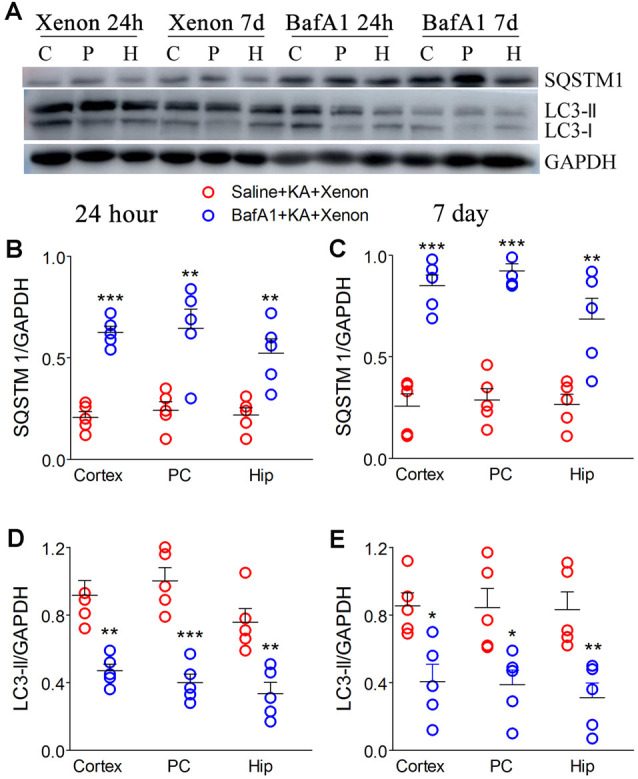
Treatment with BafA1 attenuated the increased autophagy after xenon inhalation. **(A)** Increased expression of SQSTM1 and decreased expression of LC3-II; **(B,C)** normalized intensity of SQSTM1 relative to GAPDH; **(D,E)** normalized intensity of LC3-II relative to GAPDH. Data are presented as mean ± SEM. Error bars indicate SEM (*n* = 5/group; *P < 0.05, **P < 0.01, and ***P < 0.001, compared with controls, one-way ANOVA). C, cortex except pyriform cortex; P/PC, pyriform cortex; H, hippocampus.

**Figure 6:**
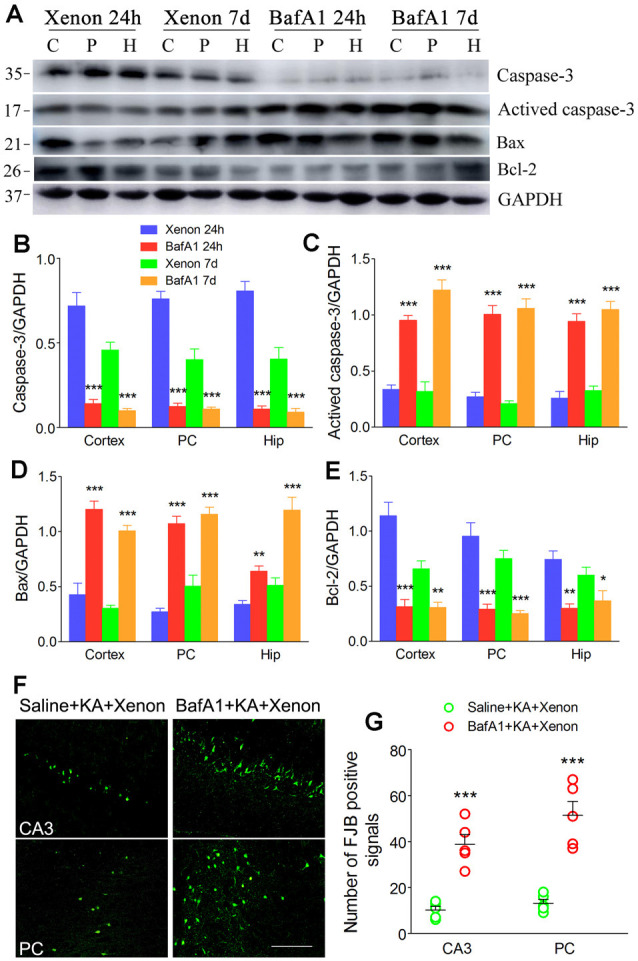
Treatment with BafA1 reversed the attenuated neuronal injury after xenon inhalation. **(A)** Increased level of activated caspase-3 and decreased level of caspase-3; **(B–E)** normalized intensity of caspase-3, activated caspase-3, Bax, and Bcl-2 relative to GAPDH; **(F,G)** FJB staining (bar = 100 μm) and account of FJB positive signals. Data are presented as mean ± SEM. Error bars indicate SEM (*n* = 5/group; *P < 0.05, **P < 0.01, and ***P < 0.001, compared with controls, one-way ANOVA). C, cortex except pyriform cortex; P/PC, pyriform cortex; H, hippocampus.

BafA1 is a proton-pump and autophagy inhibitor (Zheng et al., 2020). We injected 200 ng of BafA1 dissolved in 5 μl saline into the lateral ventricle 6 h before xenon treatment, while the control rats were instead injected with saline. The BafA1 effects were similar to those of 3-MA. BafA1 treatment significantly increased SQSTM (Figures 5A–C) and decreased LC3-II (Figures 5A,D,E) from 24 h onward. Moreover, BafA1-treated rats had significantly higher levels of activated caspase-3 (Figures 6A,C), Bax (Figures 6A,D) and lower levels of caspase-3 (Figures 6A,E) and Bcl-2 (Figures 6A,B) than those treated with saline from 24 h onwards (*n* = 5 per group). Moreover, BafA1-treated rats had a higher number of FJB-positive signals than the saline-treated control rats (Saline+KA+xenon group; the CA3 and PC; Figures 6F,G). Taken together, BafA1 reduced the autophagy level and reversed the neuroprotective effects of xenon.

### Rapamycin Promoted Autophagy and Simulated the Xenon Protective Effects

**Figure 7:**
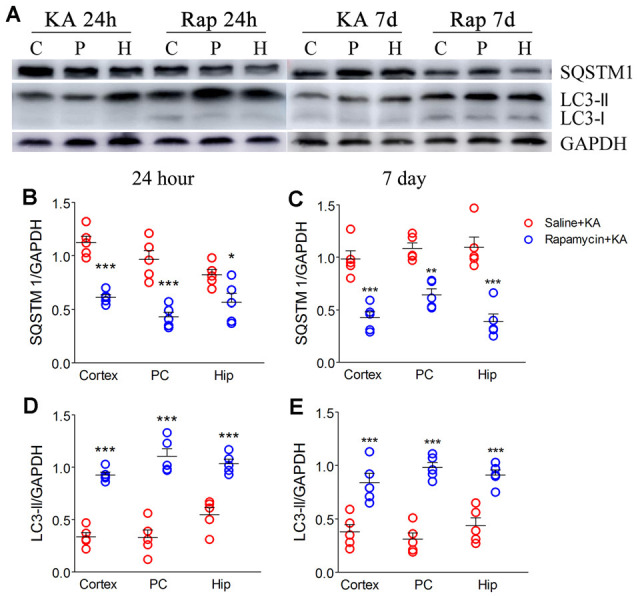
Rapamycin enhanced autophagy. **(A)** Decreased expression of SQSTM1 and increased expression of LC3-II; **(B,C)** normalized intensity of SQSTM1 relative to GAPDH; **(D,E)** normalized intensity of LC3-II relative to GAPDH. Data are presented as mean ± SEM. Error bars indicate SEM (*n* = 5/group; *P < 0.05, **P < 0.01, and ***P < 0.001, compared with controls, one-way ANOVA). C, cortex except pyriform cortex; P/PC, pyriform cortex; H, hippocampus.

**Figure 8:**
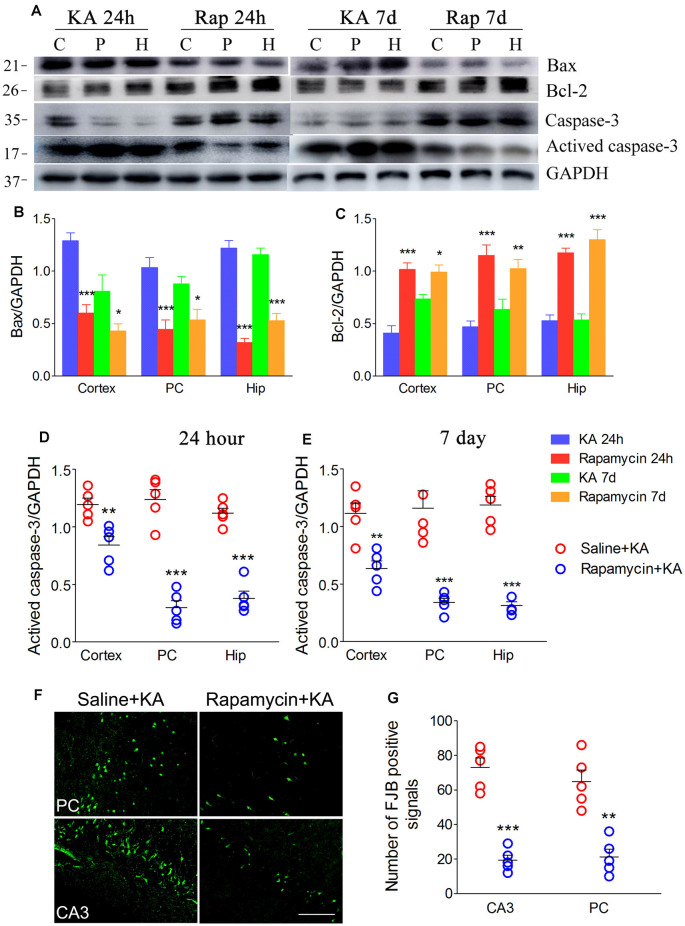
Rapamycin simulated the neuronal protective effect of xenon. **(A)** Decreased level of activated caspase-3 and increased level of caspase-3; **(B–E)** normalized intensity of caspase-3, activated caspase-3, Bax and Bcl-2 relative to GAPDH; and **(F,G)** the changes of positive FJB signal after rapamycin treatment (bar = 100 μm). Data are presented as mean ± SEM. Error bars indicate SEM (*n* = 5/group; *P < 0.05, **P < 0.01, and ***P < 0.001, compared with controls, one-way ANOVA). C, cortex except pyriform cortex; P/PC, pyriform cortex; H, hippocampus.

Rapamycin, which is an autophagy inducer (Wang et al., 2019), was administered 1 h before KA administration to simulate xenon-induced autophagy enhancement. We found that rapamycin treatment increased autophagy levels, which was indicated by decreased SQSTM1 and increased LC3-II levels (Figures 7A–E). Moreover, it attenuated apoptosis, which was indicated by decreased levels of activated caspase-3 (Figures 8A,D,E) and Bax (Figures 8A,B), and increased levels of Bcl-2 (Figures 8A,C; *n* = 5 per group). Furthermore, the FJB staining results showed that rapamycin-treated mice had a significantly lower number of FJB-positive signals than that in control rats (CA3 and PC; Figures 8F,G). Taken together, rapamycin treatment exerts a similar autophagy promotion and protective effect as that of xenon, involving both, attenuation of apoptosis and neuronal injury.

## Discussion

Previous studies have demonstrated the strong xenon neuroprotective effects (Metaxa et al., 2014; Lavaur et al., 2016a,b; Zhang et al., 2019a,b); however, the possible xenon neuroprotective effects and the underlying mechanisms in seizure-induced neuronal injury remain clear. In this study, we assessed the effects of xenon treatment on neuronal injury; moreover, we evaluated the role of autophagy in KA-induced acute generalized seizures and the involved xenon neuroprotective effects. There were no significant changes in the autophagy level with KA-induced acute generalized seizures. Contrastingly, xenon treatment significantly increased autophagy levels and attenuated neuronal injury. Moreover, the protective effects of xenon were impeded by autophagy inhibitors, including 3-MA and BafA_1_, and simulated by an autophagy inducer (rapamycin). These findings suggest that increased autophagy levels may be involved in the protective effects of xenon.

Epilepsy is characterized by neuronal over-excitation. Excessive excitation of the N-methyl-D-aspartate receptor, which results from extracellular glutamate accumulation, causes acute nerve injury or even death, by activating calpain and the caspase-3 pathway in patients with epilepsy and in animal models of epilepsy (Baudry and Bi, 2016; Hoque et al., 2016; Ceccanti et al., 2018). Xenon treatment regulates the glutamate level by suppressing its uptake and efflux (Lavaur et al., 2016a). This results in the upregulation of the anti-apoptotic protein Bcl-2 and downregulation of the pro-apoptosis protein Bax. Therefore, the xenon neuroprotective effect involves the inhibition of excessive excitation and attenuation of apoptosis (Preckel et al., 2006; Sinha and Cheung, 2010). Consistent with previous findings, we found that xenon treatment strongly prevented neuronal injury induced by KA-induced acute generalized seizures.

Increased oxidative stress resulting from over-excitation could cause autophagy (Signorelli et al., 2019). Autophagy plays an important role during apoptosis; however, its role in the pathological state remains controversial. Autophagy has been shown to play a protective role in some pathological states and its promotion is beneficial in some neurodegenerative diseases, including Parkinson’s disease and Alzheimer’s disease (Zhang et al., 2017). Contrastingly, autophagy could aggravate mitochondrial functional defects, which results in ROS accumulation and neuronal damage (Feng et al., 2020). So far, the role of autophagy in epilepsy- or seizure-induced neuronal injury remains unclear for complex seizure types and different epileptic development periods; however, previous studies have reported possible anti-epileptic effects of autophagy (Jain et al., 2016; Ni et al., 2016; Zhu et al., 2016; Kim et al., 2018). Therefore, we examined post-xenon treatment changes in molecular autophagy markers during KA-induced acute epileptic seizures to determine the involvement of autophagy in the xenon neuroprotective effect. We found that KA-induced epilepsy did not cause significant changes in autophagy markers; however, xenon inhalation significantly increased the autophagy level and attenuated neuronal injury in some brain subregions, including the PC, entorhinal cortex, and hippocampus, which are closely associated with the epilepsy development (Hsu, 2007; Petit et al., 2014; Parker et al., 2017; Sun et al., 2018). The previous reports confirmed the protection of increased autophagy in PTZ- and pilocarpine-induced seizures, though no significant changes in autophagy were found (Hosseinzadeh et al., 2016; Zhu et al., 2016). Our findings also suggest that increased autophagy levels could be closely associated with the neuroprotective effects of xenon. However, the effects of the increased autophagy in xenon treatment is unclear, because the dual roles were confirmed under different physiological or pathological conditions (Wen et al., 2008; Wang and Klionsky, 2011; Wang et al., 2011; Zhang et al., 2017; Feng et al., 2020). Moreover, whether an increased autophagy level is the underlying mechanism or is caused by the xenon neuroprotective effects remains unclear.

We administered autophagy inhibitors (3-MA and BafA_1_) to evaluate the role of autophagy in the xenon protective effects. We found that pretreatment with these autophagy inhibitors decreased the autophagy level and impeded the xenon protective effects. Contrastingly, the administration of rapamycin, an autophagy inducer, partly simulated the xenon neuroprotective effects in KA-induced acute generalized seizures. These findings further indicate that increased autophagy levels could be an underlying mechanism of the xenon neuroprotective effect and that simulating autophagy function could be a novel means of attenuating neuronal injury; however, this requires further research.

The overproduction of ROS has been confirmed in epileptic seizure (Reid et al., 2014). Due to active metabolism and oxygen consumption, mitochondria and neurons are particularly vulnerable to ROS accumulation, and injury or even death may occur due to oxidative stress. Meanwhile, the injury of mitochondria further produces even more ROS (Hattori et al., 2014; Yang et al., 2018). The damaged mitochondria could be cleaned by autophagy, ultimately, the function of mitochondria is stable (Wang and Klionsky, 2011; Lin et al., 2019), so the elevated level of autophagy attenuated ROS accumulation and neuronal injury in Parkinson’s disease and Alzheimer’s disease (Zhang et al., 2017). Similarly, our results indicate that the increased autophagy level may underlie the neuroprotective effect of xenon. Cleaning the injured mitochondria may contribute to the protection of autophagy, though some studies found that autophagy aggravated the damage of mitochondria, promote ROS production and neuronal injury in different pathological status (Wen et al., 2008; Wang et al., 2011; Feng et al., 2020).

As an inhibitor of mTOR, rapamycin has broad effects on neuronal survival, regeneration, and apoptosis. Even the effects of rapamycin on epilepsy are opposite at different intervention timepoints (Chen et al., 2012). Rapamycin intervention 10 h before KA inhibits epileptic seizure and neuronal cell death. Conversely, pretreatment with rapamycin 1 h before KA significantly promotes epileptic seizure and neuronal injury. Even rapamycin 6 h before KA results in weak aggravation. The age-, treatment paradigm-, and mode-specific anticonvulsant effects have also been confirmed (Chachua et al., 2012). These studies indicate that differences in pathological status may influence the effects of rapamycin intervention. In our preliminary experiment, the effects of rapamycin treatment 4 and 6 h before KA administration were evaluated. Significant neuronal protection was found in rats treated with rapamycin 6 h before KA. The neuroprotection difference of rapamycin at different intervention time points may be due to the different pathological development by the KA disposal method. The aggravation of rapamycin was found in KA intraperitoneal injection (12 mg/kg)-induced seizure, and seizure latency over 40 min (Chen et al., 2012). In our study, KA was administered *via* intracerebroventricular injection (3.25 × 10^–3^ mg/kg), and almost all rats had acute generalized seizures immediately after KA treatment, and even during the KA injection.

Additionally, given the crucial regulation roles of the PC and hippocampus in epileptic network development (Hsu, 2007; Petit et al., 2014; Parker et al., 2017; Sun et al., 2018), the neuroprotection of rapamycin in vital brain regions is most likely influence the epileptic development than acute seizure (Chachua et al., 2012).

## Conclusions

In summary, this study provides evidence of the strong neuroprotective effect of xenon during KA-induced acute generalized seizures. Moreover, our findings indicate that increased autophagy levels might be involved in the xenon neuroprotective effect. Therefore, proper autophagy activation may be an effective approach for preventing seizure-induced neuronal injury.

## Data Availability Statement

All datasets presented in this study are included in the article/Supplementary Material.

## Ethics Statement

The animal study was reviewed and approved by Binzhou Medical University Animal Experimentation Committee (Approval No. 2018002).

## Author Contributions

WZ: study conception and design and data acquisition. JZ, SZ, JL, YZ, DH, and HS: KA-induced rat model preparation, data acquisition, data analysis, and interpretation. All authors contributed to the article and approved the submitted version.

## Conflict of Interest

The authors declare that the research was conducted in the absence of any commercial or financial relationships that could be construed as a potential conflict of interest.

## Supplementary Material

SUPPLEMENTARY FIGURE S1 Details on the experimental procedure.

## Abbreviations

3-MA: 3-methyladenine
BafA_1_: bafilomycin A_1_
Bax: Bcl-2-associated X protein
Bcl-2: B cell lymphoma-2
EC: entorhinal cortex
GAPDH: glyceraldehyde-3-phosphate dehydrogenase
KA: kainic acid
LC 3: microtubule-associated protein 1 light chain 3
PC: pyriform cortex
SQSTM 1: sequestosome 1.
