# Supplementary figures and images for: Xenon Exerts Neuroprotective Effects on Kainic Acid-Induced Acute Generalized Seizures in Rats via Increased Autophagy
Source: Front Cell Neurosci. 2020 Oct 6;14:582872. doi: 10.3389/fncel.2020.582872 (PMC7573545; doi:10.3389/fncel.2020.582872)

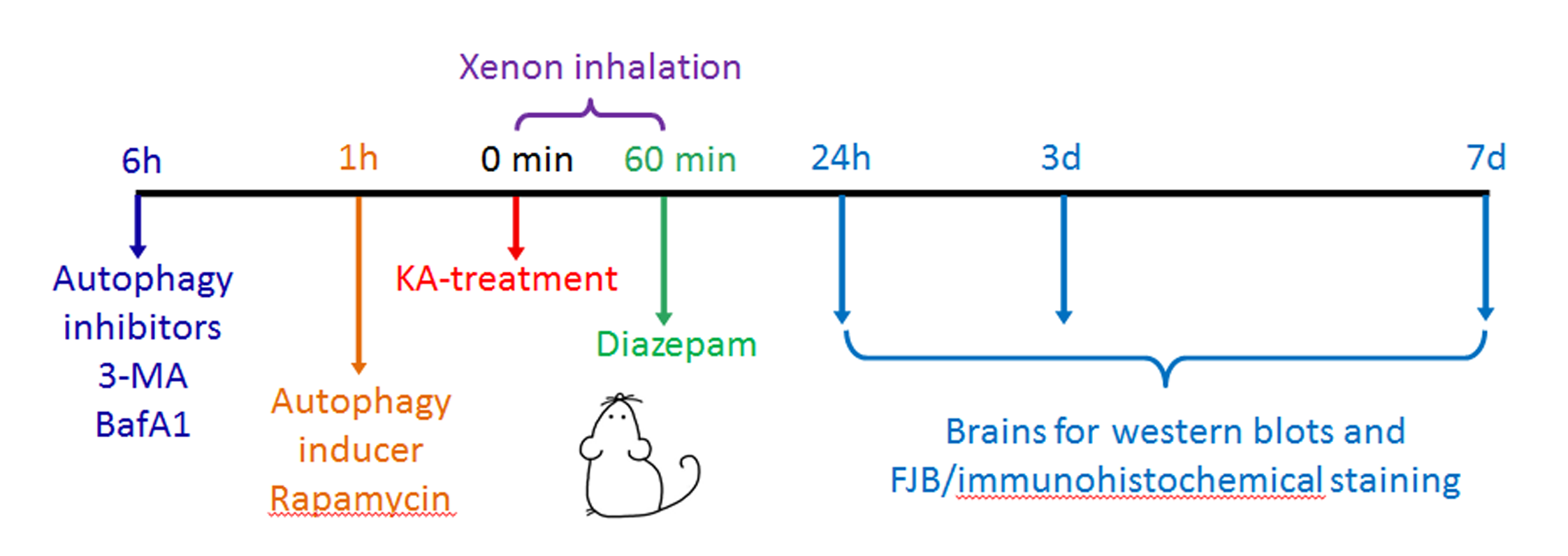

Supplement: SUPPLEMENTARY FIGURE S1 — Details on the experimental procedure. [file Image_1.tif]
